# Risk for cardiovascular disease development in rheumatoid arthritis

**DOI:** 10.1186/s12872-024-03963-3

**Published:** 2024-06-04

**Authors:** Dražen Bedeković, Ivica Bošnjak, Ines Bilić-Ćurčić, Damir Kirner, Sandra Šarić, Srđan Novak

**Affiliations:** 1 Department of Cardiovascular Diseases, Internal Medicine Clinic, University Hospital Osijek, J. Huttlera 4, Osijek, 31000 Croatia; 2 Faculty of Medicine Osijek, Department of Internal Medicine, University J.J. Strossmayer, J. Huttlera 4, Osijek, 31000 Croatia; 3 Department for Pharmacology, Faculty of Medicine, J. J. Strossmayer University of Osijek, J. Huttlera 4, Osijek, 31000 Croatia; 4 Department of Endocrinology and Metabolism Disorders, Internal Medicine Clinic, University Hospital Centre Osijek, J. Huttlera 4, Osijek, 31000 Croatia; 5 Department of Rheumatology and Clinical Immunology, University Hospital Rijeka, Rijeka, Croatia; 6 Faculty of Medicine Rijeka, Department of Internal Medicine, University of Rijeka, Braće Branchetta 20/1, Rijeka, 51000 Croatia

**Keywords:** Rheumatoid arthritis, Osteoarthritis, Chronic inflammation, Cardiovascular risk, Cardiovascular mortality

## Abstract

**Background:**

Patients with rheumatoid arthritis have significant cardiovascular mortality and morbidity.

**Objective:**

To investigate the effects of chronic inflammation in rheumatoid arthritis on cardiovascular morbidity association with cardiovascular risk factors risk factors. Mortality report is secondary just to show trends without sufficient statistical power as it is accidental endpoint.

**Methods:**

A total of 201 individuals without previous cardiovascular disease, 124 with rheumatoid arthritis (investigation group) and 77 with osteoarthritis (control group), were included in the study and followed up for an average of 8 years to assess the development of fatal or non-fatal cardiovascular diseases. The incidence and prevalence of cardiovascular risk factors were also investigated.

**Results:**

The total incidence of one or more fatal or nonfatal cardiovascular events was 43.9% in the investigation group and 37.5% in the control group. Of these patients, 31.7% and 30.9% survived cardiovascular events in the investigation and control groups, respectively. The most common cardiovascular disease among participants who completed the study and those who died during the study was chronic heart failure. The results of the subgroup analysis showed that strict inflammation control plays a central role in lowering cardiovascular risk.

**Conclusion:**

A multidisciplinary approach to these patients is of paramount importance, especially with the cooperation of immunologists and cardiologists for early detection, prevention, and management of cardiovascular risks and diseases.

## Introduction

Rheumatoid arthritis (RA) is chronic, systemic inflammatory disease that affects approximately 1% of the population, predominantly women. RA significantly increases morbidity and mortality and shortens patient lifespan by 5–18 years, mainly due to an increased incidence of cardiovascular diseases (CVD) [1–12]. Although mortality in patients with RA has decreased in recent years, several studies and meta-analyses still indicate an increased cardiovascular risk and a higher rate of adverse events, especially fatal myocardial infarction and stroke [5–7, 10, 13–16]. Meta-analysis of 14 observational studies comprising 41 490 participants found a 48% increased risk of incident CVD in patients with RA (relative risk [RR], 1.48; 95% CI, 1.36–1.62), 68% and 41% increased risks of myocardial infarction and stroke respectively, as well as an increased risk of chronic heart failure (RR, 1.87; 95% CI, 1.47–2.39), which was assessed in only one study [8].

**Fig. 1:**
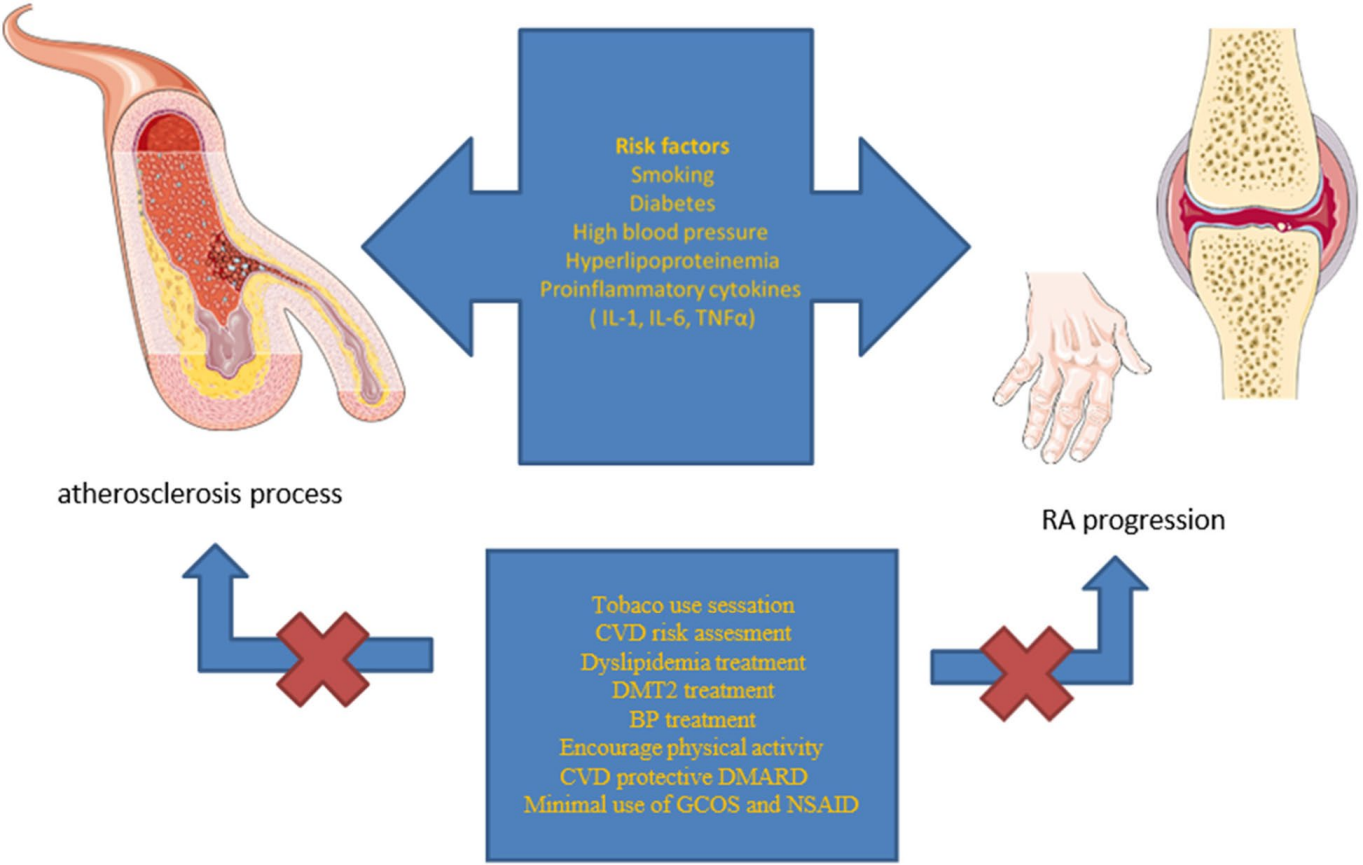
Influence of risk factors on disease progression. Legend: CVD- cardiovascular disease, DMT2-diabetes mellitus typ 2, BP- blood pressure, DMARD- disease modifying antirheumatic drug, GCOS- glucocoriticoides. NSAID-nonsteroidal antiinflammatory drugs

CV risk estimation is more difficult in patients with RA because of the combination of traditional and RA-specific risk factors. Traditional risk factors include 1) modifiable risk factors, namely arterial hypertension, dyslipidemia, insulin resistance and diabetes, cigarette smoking, low physical activity; and 2) non-modifiable risk factors such as family heritage and genetics, race, age, and sex (Fig. 1) [17]. In RA, chronic inflammation independently increases CV risk [4, 18].

Assessing CV risk using standard calculation models (the Framingham Risk Score and Systematic Coronary Risk Evaluation [SCORE], validated for RA) underestimates CV risk in low- and intermediate-risk groups and overestimates it in high-risk groups when modified models are used [16, 19–26]. SCORE2 and SCORE2-OP for older persons estimate the risk of fatal and non-fatal CV events in people aged 40–69 years with no known CVD, and different values are assigned for low-, moderate-, high-, and very high-risk countries [27–29]. Because RA increases CV risk in addition to other risk factors, studies have recommended that the calculated relative risk is multiplied by 1.4 for men and 1.5 for women if two of the three following RA criteria are met: (1) disease duration > 10 years, (2) rheumatoid factor (RF) or anti-citrulline antibody (anti-CCP) positivity, and (3) the presence of extra-articular manifestations (Felty’s syndrome, pericarditis, pleuritis, polyneuropathy, mononeuritis, episcleritis, glomerulonephritis, or skin or other organ vasculitis) [27–30]. Despite SCORE modifications, limitations to CV risk estimation and early atherosclerosis detection remained [31]. The lipid paradox and effect of inflammation on lipid particle composition in RA make the interpretation of circulating lipid levels less reliable for use in CV risk prediction models [32]. More efficient new therapies with fewer extra-articular manifestations can also affect risk estimation [33]. High-grade uncontrolled inflammation leads to accelerated atherosclerosis and early vascular damage, which directly increases CV risk and early CVD manifestations [32, 34]. The cumulative time spent in RA flares significantly impacts CV risk; Myasoedova et al. showed that CV risk was higher in patients with RA who spent more time in medium (hazard ratio [HR] 1.08; 95% CI, 0.98–1.20) and high disease activity (HR 1.18; 95% CI, 1.06–1.31) than in those with more time in lower activity [35]. According to data from the QResearch database of 2.3 million people, chronic inflammation has an influential effect on modifiable risk factors, further increasing CV risk [36]. A recent meta-analysis by Wang et al. of 1446 patients and 205 575 controls reported CV outcomes in patients with coronary heart disease with and without RA and showed that all-cause mortality (RR, 1.47; 95% CI, 1.34–1.61; *p* = 0.00001), cardiac death (RR, 1.51; 95% CI, 1.05–2.17; *p* = 0.03) and congestive heart failure risk (RR, 1.41; 95% CI, 1.27–1.56; *p* = 0.00001) were significantly higher in patients with RA; further, the risks for myocardial infarction, repeated revascularization, and percutaneous coronary intervention were similar in patients with and without RA [37]. Analysis of the Japanese health insurance database revealed a significantly increased risk of ischemic heart disease and cerebral infarction in patients with RA [2]. Biomarkers have not been used in risk calculations although they could have sensitive prediction of CVD like well-known N-terminal pro-brain natriuretic peptide (NT – pro BNP) [38, 39], or novel biomarkers as: Galectin-3, impaired endogenous fibrinolysis, trimethyllysine and trimethylamine N-oxide, and serum cholesterol efflux capacity were but further evaluation of their prognostic value in is needed [40–44]. The use of non-invasive diagnostic methods such as carotid ultrasound or multi-slice computed tomography (MSCT) in patients with a calculated intermediate CVD risk may improve risk stratification [15, 45]. A meta-analysis of studies that measured the coronary calcium score using MSCT indicated a higher prevalence of asymptomatic coronary disease, multivessel disease, and high-risk plaques in patients with RA than in controls [45, 46]. According to observational analyses by Gossec et al. and the COMORA study of comorbidities in RA, the screening assessment of CVD by physicians remains insufficient, and approximately 70–90% of patients with RA are unaware of increased CV risk, especially in those with a greater number of traditional CV risk factors [47–50].

RA therapy with certain medications such as glucocorticoids and nonsteroidal antirheumatic drugs can also contribute to increased CV risk, although methotrexate and tumor necrosis factor alpha inhibitors may have beneficial effects [49].

In osteoarthritis (OA), there is an increased prevalence of CVD and CV risk of approximately 55% compared with that in the general population, although chronic inflammation is not OA leading cause. Onset of OA usually lather than RA where ageing and accumulation of other CV risk factors play significant role in CVD development [51].

In this study, we hypothesized that uncontrolled chronic inflammation contributes significantly to CV risk, morbidity, and mortality. This investigation was dedicated to addressing the problem of earlier development of CVD and CV risk factors in patients with RA in Eastern Croatia.

## Patients and methods

A total of 201 patients were recruited for the prospective cohort study from the Rheumatology Center of the University Hospital Osijek: 124 diagnosed with RA (investigation group) and 77 with OA (control group). The participants underwent an average observational period of 8 years and 4 months ± 3 months between 2008/9 and 2016/17. In selection process patients diagnosed with any form of CVD, including incidental findings of CVD on diagnostic tests or symptomatic heart failure were excluded. CVD included previously diagnosed: any type of myocardial infarction, any type of angina pectoris, coronary disease proven on diagnostic tests, symptomatic or asymptomatic ischemic cardiomyopathy, stroke or transient ischemic attack, acute or chronic dissection or aneurysm of the aorta, symptomatic or asymptomatic peripheral arterial disease. Any patient suspected on CVD based on recorded or detected signs, symptoms or tests was also excluded (chest pain, shortness of breath and exertion intolerance, syncope, electrocardiogram (ECG) changes, treadmill test etc.). Coronary angiography confirmed or excluded epicardial artery disease.

Heart failure was diagnosed according existence of signs and symptoms: breathlessness, ankle swelling, and chronic fatigue without non-cardiac cause, elevated jugular venous pressure – prominent jugular veins, pulmonary crackles not related to pulmonary diseases, bilateral peripheral edema or abnormal ECG found recorded in patient’s charts or at initial examination. At recruitment time echocardiography availability was very limited in our hospital and specific laboratory tests were not available so we had to rely on medical records, signs and symptoms and ECG for heart failure for exclusion. But as laboratory parameters (BNP, NT-pro BNP) and echocardiography become available during follow up every participant suspected for heart failure went through diagnostic process an if criteria according to European Society of Cardiology (ESC) guidelines were met was diagnosed and treated for heart failure (reduced, mild range or preserved left ventricle ejection fraction) [17].

RA or OA was diagnosed by a rheumatologist according to the American College of Rheumatology classification criteria. All the participants provided written informed consent to participate in this study. The inclusion criterion was also permanent residence in one of the five counties in eastern Croatia. Participants who migrated or declined to participate during the study period were excluded. All participants (investigation and control group) attended the initial (inclusion) and final visits (if available), and patients from the investigation group attended annual visits to assess RA activity. At initial visit all participants went through pre-prepared extensive questionnaire, physical exam and tests. If participant was suspected to any cardiovascular disease additional appropriate tests were made at any visit to confirm or exclude it.

During investigation period participants were continually monitored for any changes in their health status, especially for occurrence of CVD: myocardial infarction (STEMI, NSTEMI), unstable or stable angina pectoris, coronary disease found on diagnostic tests (epicardial artery or microvascular disease), ischemic cardiomyopathy and heart failure, stroke, transient ischemic attack, acute or chronic dissection or aneurysm, penetrating aortic ulcer or aortic intramural hematoma peripheral arterial disease, or new CV risk factor, occurrence of infections or neoplastic diseases, surgeries, or RA suspected od confirmed flare, changes in any medicament therapy or introduction of new medicament (list was provided). Participants could contact investigators by telephone, mail, e-mail, or personal visits, and for majority of participants compliance was excellent. Because of we are the only rheumatology center in region we as well as the only hospital with cardiology with percutaneous coronary intervention capability, cardiac and vascular surgery departments and neurology department capable of treating acute diseases we used computerized integrated hospital information system for monitoring. If participant was hospitalized at any department or went to emergency unit we investigated if cardiovascular etiology was possible cause of condition. We made periodical contact with participants family physicians to confirm od add information because they are obligated to have patient personal records were everything regarding health is recorded from birth until death. Finally, at final visit all participants went thorough questionnaire; information about CVD, CV risk factors, therapy, and other investigation parameters were thoroughly checked and physical exam and tests were done. After final visit for additional check we made visits to participant’s family physicians who provided us insight to participants records. If the participant died, the cause of death, comorbidities and known cardiovascular risk factors diagnosed before death were recorded. Data were obtained from autopsies, coroners’ reports, reports from the Croatian Institute for Public Health, hospitals, and primary care physician records.

Investigation methods comprised completion by the RA and OA groups of a pre-prepared questionnaire written in Croatian; physical examination with measurement of blood pressure three times in 10 min apart and taking average value as final, body weight and height, and waist and hip circumference; assessment of general health and pain intensity using visual-analog scales; completion of the DAS28-CRP (Disease Activity Score 28 with C-reactive protein; RA group), arthritis severity index for OA of the hand (OA group), Lequesne index for OA of the hip and knee (OA group); completion of the Croatian translation of the Health Assessment Questionnaire (RA and OA groups); examination using a 12-channel electrocardiogram (ECG); and collection of venous blood for laboratory analysis of erythrocyte sedimentation rate, C-reactive protein (CRP), total cholesterol, high density lipoprotein (HDL), low density lipoprotein (LDL), triglycerides, creatinine, blood glucose, and glycosylated hemoglobin (HbA1c; RA and OA groups). If indicated, an oral glucose load test was also performed. Finally, RF and anti-CCP levels were measured in patients with RA. According to our laboratory pathologic test results were: CRP > 5 mg/L, total cholesterol > 5 mmol/L, HDL < 1.03 mmol/L for male and < 1.29 mmol/L for female participants, LDL > 3 mmol/L, triglycerides > 1.7 mmol/L, creatinine > 90 mmol/L, fasting blood glucose > 6.3 mmol/L, HbA1c > 6.5%, RF > 15 mlU/ml, anti-CCP > 25 u/ml. DAS28-CRP score criteria were: remission < 2.6, low activity > 2.6 < 3.2, moderate activity > 3.2 < 5.1 and high activity > 5.1. All forms were completed by a physician based on the information obtained.

For RA inflammation control subgroup analysis we accepted participants who did not missed more than two annual visits, what was minimum requirement to asses disease activity, however majority of participants had in average 3 annual visits mostly due to suspected or confirmed flare where disease activity was assessed. Participants were classified in remission group if had low RA activity more than 60% of study time, otherwise were classified in unsatisfactory disease control. Acute infectious disease or other disease which increase inflammation markers was not classified as RA flare.

### Statistical analyses

Descriptive statistics were used to describe and summarise the study data. Inferential statistics were applied to test the hypotheses. Independent samples t-test was employed to check the significance of mean differences. Depending on Levene’s test results, the t-test assuming equal or unequal variance was used to determine statistical significance between groups. A chi-square test of independence was applied to analyse the relationship between qualitative variables. Fisher's exact test was employed when assumptions for the chi-square test were not met. The level of significance was set at *p* < 0,05, whereas 0,05 < *p* < 0,10 was considered as a tendency (marginally significant).

## Results

At the first visit in 2008/9, after a thorough selection process, 201 participants were included in the study, comprising 124 with RA and 77 with OA. At the final visit in 2016/17, 137 participants (82 with RA and 55 with OA) remained enrolled. A total of 58 patients, 41 with RA and 17 with OA, died during the study period. An additional six individuals (one with RA and five with OA) declined to participate further in the study or left the country. The investigation (RA) group initially included 25 men and 99 women, whereas the control (OA) group comprised 11 men and 66 women. The average age was 59.78 (37–81) years in the RA group and 64.23 (27–80) years in the OA group, OA participants were significantly older (*p* = 0,004). The average disease duration was significantly longer, 12.2 years, for the RA group than in 5.64 years for the OA group (*p* = 0,001). For RA group DAS28-CRP was an average of 4.94 (standard deviation 1.41, range 1.46–8.08), which reflected moderate disease activity, and 82.3% were RF-positive. At the final visit, 137 patients were available for analysis (82 with RA [16 men and 66 women] and 55 with OA [10 men and 45 women]), with an average age of 65.5 years in the RA and 71.2 years in the OA group. For RA group DAS28-CRP averaged 4.08 (standard deviation 1.12, range 1.38–6.34) which reflected moderate disease activity, and 90.2% were RF-positive.

The use of glucocorticoids in the RA group was continuously high during the investigation (74.2% and 76.8% at the initial and final visits, respectively; *p* = 0.332), as well as the prevalence of painkiller use was continuously high in both groups. Usage of any painkillers at the initial visit was 87.1% in RA and 90.9% in OA (*p* = 0.41), while at the final visit usage was 95.1% for RA and 89.1% for OA (*p* = 0.55), NSAIDs usage at initial visit was 55.1% for RA and 63.9% for OA (marginally significant difference; *p* = 0.051), while at the final visit was 75.4% for RA and 60% for OA (*p* = 0.17).

At the first visit participants with hypertension 77,4% (RA group) and 91,7% (OA group) (*p* = 0.039) used one or more anti-hypertensives, while at final visit usage was 91,7 and 95,5% (*p* = 2.257) for RA and OA group, respectively. Use of statins at first visit was 23,3% (RA), 25,4% (OA) (*p* = 8.857), and at final visit 30,7% (RA), 40% (OA) (*p* = 0.850) for participants with hypercholesterolemia. Of those LDL in referral values had 37,5% (RA) and 66,7% (OA) (*p* = 0.11) at first visit and 43,5% and 38,9% at final visit (*p* = 1) for RA and OA groups respectively.

The total incidence of one or more cardiovascular events or death from CVD was 43.9% in the RA group and 37.5% in the OA group; of these, 31.7% in the RA and 30.9% in the OA group survived event. The most common cardiovascular diagnosis among the participants who completed the study and those who died during the study was chronic heart failure. Acute CVD was much less frequent in both living and deceased participants (Table 1). The total number of participants who died was surprisingly high for RA (33.1%, 41 of 124 participants) and OA (22.1%, 17 of 77 participants). CVD was the cause of 70.7% of deaths in the RA group and 58.8% in the OA group (Table 2).

**Table 1:** The incidence of cardiovascular disease; non-fatal and fatal + non-fatal cumulative

Incidence (%)	Rheumatoid arthritis group—Visit 20016/17 (*N* = 82)	Osteoarthritis group—visit 2016/17 (*N* = 55)
CARDIOVASCULAR DISEASE FROM THE 1. VISIT—ALIVE	31,7	30,9
ARTERIAL HYPERTENSION FROM THE 1. VISIT—ALIVE	17,1	12,7
HEART DISEASE TOTAL FROM 1. VISIT -ALIVE	31,7	38,2
HEART FAILURE TOTAL FROM 1. VISIT—ALIVE	14,6	12,7
PERIPHERAL ARTERY DISEASE FROM THE 1. VISIT—ALIVE	2,4	3,6
TRANSIENT ISCHEMIC ATTACK FROM THE 1. VISIT—ALIVE	4,9	3,6
STROKE FROM 1. VISIT—ALIVE	4,9	9,1
ACUTE MYOCARDIAL INFARCTION SINCE THE 1. VISIT—ALIVE	6,1	7,3
ANGINA PECTORIS LIVES FROM THE 1. VISIT—ALIVE	7,3	9,1
CORONARY DISEASE—OTHER FORMS OF THE 1. VISIT—ALIVE	2,4	3,6
AORTIC ANEURYSM FROM THE 1. VISIT—ALIVE	0,0	1,8
SEVERE INFECTION FROM 1. VISIT—ALIVE	9,8	9,1
DIED OF CARDIOVASCULAR DISEASE	70,7 (*N* = 41)	58,8 (*N* = 17)
TOTAL CARDIOVASCULAR DISEASE (ALIVE + DECEASED)	43,9 (*N* = 123)	37,5 (*N* = 72)
TOTAL STROKE (ALIVE + DECEASED)	4,9 (*N* = 123)	11,1 (*N* = 72)
TOTAL MYOCARDIAL INFARCTION (ALIVE + DECEASED)	4,9 (*N* = 123)	9,7 (*N* = 72)
TOTAL AORTIC ANEURYSM (ALIVE + DECEASED)	1,6 (*N* = 123)	1,4 (*N* = 72)

**Table 2:** Deceased incidence and cardiovascular risk factor prevalence among deceased

Deceased prevalence (%)	Rheumatoid arthritis (*N* = 41)	Osteoarthritis (*N* = 17)	Total (*N* = 58)
ARTERIAL HYPERTENSION	80,5	70,6	77,6
HYPERCHOLESTEROLEMIA	82,9	70,6	79,3
DIABETES MELLITUS	29,3	41,2	32,8
CIGARETTE SMOKING	36,6	29,4	34,5
MALE GENDER	22	17,6	20,7
FEMALE GENDER	78	82,4	79,3
DEATH INCIDENCE (%)			
TOTAL DEAD	33,1 (*N* = 124)	22,1 (*N* = 77)	28,9 (*N* = 201)
DIED OF CARDIOVASCULAR DISEASE	70,7 (*N* = 41)	58,8 (*N* = 17)	65,5 (*N* = 58)

We compared our findings with 2018. Croatian general population data. Of the 41 participants who died of RA, CVD was the cause for 28 (70.7%). In the population data, CVD was the cause of death for 23 504 of 53 477 people (44%). The z-test revealed a statistically significant difference in the proportion of CVD deaths (z = 3.139, *p* = 0.002). In the OA group, 10 of the 17 deceased patients died from CVD (58.8%). The z-test showed no significant difference in the proportion of CVD deaths between the OA and population groups (z = 1.235, *p* = 0.217) [52].

Generally, the prevalence of CV risk factors in both study groups was high, particularly that for arterial hypertension and increased cholesterol. However, these mostly did not differ among the groups, with a few exceptions. The cumulative number of CV risk factors steadily increased over time (Table 3).

**Table 3:** The prevalence of risk factors for cardiovascular disease for participants who completed study and deceased

Prevalence (%)	Rheumatoid arthritis group—visit 2008/9 (*N* = 124)	Osteoarthritis group—visit 2008/9 (*N* = 77)	Rheumatoid arthritis group—visit 20016/17 (*N* = 82)	Osteoarthritis group—visit 2016/17 (*N* = 55)
MALE GENDER	20,2	14,3	19,5	18,2
FEMALE GENDER	79,8	85,7	80,5	81,8
USE OF GLUCOCORTICOIDS	74,2		76,8	
ARTERIAL HYPERTENSION	62,1	68,8	73,2	80,0
SMOKING CIGARETTES EVER	46,8	27,3	52,4	25,5
SMOKING CIGARETTES NOW	21,8	14,3	29,3	10,9
DIABETES MELLITUS	16,1	16,9	32,9	29,1
HYPERCHOLESTEROLEMIA	83,1	81,8	91,5	81,8
METABOLIC SYNDROME	57,3	67,5	62,2	65,5
NUMBER OF RISK FACTORS OF CORONARY DISEASE 0	5,6	3,9	2,4	1,8
NUMBER OF RISK FACTORS OF CORONARY DISEASE 1	26,6	29,9	9,8	29,1
NUMBER OF RISK FACTORS OF CORONARY DISEASE 3	20,2	15,6	29,3	27,3
NUMBER OF RISK FACTORS OF CORONARY DISEASE 4	0,0	1,3	6,1	3,6
HEART DISEASES TOTAL / NON-ISCHEMIA FOR 2008, ALL FOR 2016	16,1	16,1	37,8	49,1
ARRHYTHMIAS TOTAL	8,9	7,8	19,5	18,2
ATRIAL FIBRILLATION	3,2	3,9	8,5	10,9
ARTERIAL HYPERTENSION DECEASED			80,5 (*N* = 41)	70,6 (*N* = 17)
HYPERCHOLESTEROLEMIA DECEASED			82,9 (*N* = 41)	70,6 (*N* = 17)
DIABETES MELLITUS DECEASED			29,3 (*N* = 41)	41,2 (*N* = 17)
CIGARETTE SMOKING DECEASED			36,6 (*N* = 41)	29,4 (*N* = 17)
MALE DECEASED			22 (*N* = 41)	17,6 (*N* = 17)
FEMALE DECEASED			78 (*N* = 41)	82,4 (*N* = 17)
ARTERIAL HYPERTENSION (ALIVE + DECEASED)			75,6 (*N* = 123)	77,8 (*N* = 72)
DIABETES MELLITUS (ALIVE + DECEASED)			31,7 (*N* = 123)	31,9 (*N* = 72)
HYPERCHOLESTEROLEMIA (ALIVE + DECEASED)			88,6 (*N* = 123)	79,2 (*N* = 72)
CIGARETTE SMOKING (ALIVE + DECEASED)			47,2 (*N* = 123)	26,4 (*N* = 72)

At the first (inclusion) visit, the RA group had significantly lower body mass index (BMI) and triglyceride levels, marginally higher systolic and diastolic blood pressure, and a significantly larger proportion of former and current cigarette smokers. Other parameters did not differ significantly (Tables 4 and 5).

**Table 4:** Association of cardiovascular risk between groups at inclusion visit

RA-OA 2008/9	Statistically significant difference	χ2	p
PREVALENCE OF HYPERTENSION	NO	0,943	0,332
PREVALENCE OF SMOKING EVER	YES—RA HAS HIGHER	7,573	0,006
PREVALENCE OF SMOKING NOW	NO	1,737	0,187
PREVALENCE OF DIABETES	NO	0,02	0,888
PREVALENCE OF HIGH TOTAL CHOLESTEROL	NO	0,051	0,821
PREVALENCE OF METABOLIC SYNDROME	NO	2,112	0,146
PREVALENCE OF AVERAGE NUMBER OF CARDIOVASCULAR RISK FACTORS	NO		0,677
PREVALENCE OF HEART DISEASE—NOT CARDIOVASCULAR	NO	1,628	0,202
PREVALENCE OF ARRHYTHMIAS	NO	0,071	0,789
PREVALENCE OF ATRIAL FIBRILLATION	NO		1

**Table 5:** Prevalence of cardiovascular risk factors at inclusion visit

**Group**		**N**	**Mean**	**Std. Deviation**	**Std. Error Mean**	**Statistically significant difference**	**t**	**p**
SYSTOLIC BP	RHEUMATOID ARTHRITIS	124	136,96	17,47	1,57	NO—MARGINAL RA HAS HIGHER	1,735	0,084
	OSTEOARTHRITIS	77	133,17	13,35	1,52			
DIASTOLIC BP	RHEUMATOID ARTHRITIS	124	84,30	9,19	0,83	NO—MARGINAL RA HAS HIGHER	1,961	0,051
	OSTEOARTHRITIS	77	81,82	7,89	0,90			
BMI	RHEUMATOID ARTHRITIS	124	28,00	5,88	0,53	YES RA HAS LOWER	-2,58	0,011
	OSTEOARTHRITIS	77	30,07	4,96	0,57			
WAIST- HIP RATIO	RHEUMATOID ARTHRITIS	124	0,91	0,10	0,01	NO	0,638	0,524
	OSTEOARTHRITIS	77	0,90	0,08	0,01			
HAQ	RHEUMATOID ARTHRITIS	124	1,65	0,93	0,08	YES—RA HAS HIGHER	2,438	0,016
	OSTEOARTHRITIS	77	1,40	0,55	0,06			
VAS	RHEUMATOID ARTHRITIS	124	6,02	2,46	0,22	NO	0,712	0,477
	OSTEOARTHRITIS	77	5,77	2,34	0,27			
GH	RHEUMATOID ARTHRITIS	124	43,42	25,39	2,28	NO	-0,237	0,813
	OSTEOARTHRITIS	77	44,16	18,47	2,10			
ESR	RHEUMATOID ARTHRITIS	122	35,34	25,06	2,27	YES—RA HAS HIGHER	5,532	0,001
	OSTEOARTHRITIS	74	19,70	14,49	1,68			
TOTAL CHOLESTEROL	RHEUMATOID ARTHRITIS	124	5,85	1,24	0,11	NO	-0,39	0,697
	OSTEOARTHRITIS	77	5,93	1,38	0,16			
HDL	RHEUMATOID ARTHRITIS	123	1,53	0,46	0,04	NO	0,395	0,693
	OSTEOARTHRITIS	75	1,48	1,40	0,16			
LDL	RHEUMATOID ARTHRITIS OSTEOARTHRITIS	123 72	3,57 3,89	0,99 3,52	0,09 0,42	NO	-0,716	0,449
TRIGLYCERIDE	RHEUMATOID ARTHRITIS	124	1,65	0,70	0,06	YES—RA HAS LOWER	-3,46	0,001
	OSTEOARTHRITIS	77	2,22	1,34	0,15			
CREATININE	RHEUMATOID ARTHRITIS	124	79,03	19,95	1,79	NO	0,971	0,408
	OSTEOARTHRITIS	75	76,69	18,16	2,10			
BLOOD GLUCOSE	RHEUMATOID ARTHRITIS	124	5,68	1,89	0,17	NO	-0,976	0,33
	OSTEOARTHRITIS	77	5,96	2,22	0,25			

*Legend*: *BP* blood pressure, *BMI* body mass index, *HAQ* health assessment questionnaire, *VAS* visual-analog scale, *GH* general health, *ESR* erythrocyte sedimentation rate, *HDL* high density cholesterol, *LDL* low density cholesterol

At the final visit, the RA group had significantly higher HDL levels than the OA group; however, no other significant differences were identified. The proportion of former and current cigarette smokers was greater in the RA group throughout the study, and the prevalence of high cholesterol and total number of CV risk factors was also consistently higher in the RA group (Tables 6 and 7).

**Table 6:** Association of cardiovascular risk factors at final visit

RA-OA 2016/17	Statistically significant difference	χ2	p
INCIDENCE OF CARDIOVASCULAR DISEASE	NO	0,01	0,912
PREVALENCE OF HYPERTENSION	NO	0,84	0,359
INCIDENCE OF HYPERTENSION	NO	0,479	0,489
PREVALENCE OF SMOKING EVER	YES—RA HAS HIGHER	9,866	0,002
PREVALENCE OF SMOKING	YES—RA HAS HIGHER	6,488	0,011
PREVALENCE OF DIABETES	NO	0,255	0,635
PREVALENCE OF HIGH TOTAL CHOLESTEROL	NO—MARGINAL RA HAS HIGHER	2,818	0,093
PREVALENCE OF METABOLIC SYNDROME	NO	0,151	0,698
PREVALENCE OF AVERAGE NUMBER OF RISK FACTORS FOR CVD	NO—MARGINAL RA HAS MORE		0,052
PREVALENCE OF HEART DISEASE—CVD + OTHER	NO	1,718	0,19
INCIDENCE OF HEART DISEASE	NO	0,612	0,434
INCIDENCE OF PERIPHERAL VASCULAR DISEASE	NO		1
INCIDENCE OF TRANSIENT ISCHEMIC ATTACK	NO		1
INCIDENCE OF STROKE	NO		0,484
INCIDENCE OF ACUTE MYOCARDIAL INFARCTION	NO		1
INCIDENCE OF ANGINA PECTORIS	NO		0,755
INCIDENCE OF OTHER FORMS OF CORONARY DISEASE	NO		1
INCIDENCE OF ANEURYSM ABD. AORTA	NO		0,401

**Table 7:** Prevalence of cardiovascular risk factors at final visit

**Group**		**N**	**Mean**	**Std. deviation**	**Std. error mean**	**Statistically significant difference**	**t**	**p**
SYSTOLIC BP	RHEUMATOID ARTHRITIS	82	131,84	18,01	1,99	NO	-0,296	0,758
	OSTEOARTHRITIS	55	132,71	14,94	2,01			
DIASTOLIC BP	RHEUMATOID ARTHRITIS	82	80,82	9,41	1,04	NO	0,25	0,803
	OSTEOARTHRITIS	55	80,40	9,84	1,33			
BMI	RHEUMATOID ARTHRITIS	82	27,90	5,42	0,60	NO	-1,482	0,141
	OSTEOARTHRITIS	55	29,27	5,16	0,70			
WAIST- HIP RATIO	RHEUMATOID ARTHRITIS	82	0,91	0,08	0,01	NO	-1,009	0,315
	OSTEOARTHRITIS	55	0,92	0,09	0,01			
HAQ	RHEUMATOID ARTHRITIS	82	1,60	0,87	0,10	NO	0,444	0,658
	OSTEOARTHRITIS	55	1,53	0,76	0,10			
VAS	RHEUMATOID ARTHRITIS	80	4,96	2,38	0,27	NO	-0,131	0,896
	OSTEOARTHRITIS	55	5,02	2,50	0,34			
GH	RHEUMATOID ARTHRITIS	80	41,54	22,17	2,48	NO	0,285	0,776
	OSTEOARTHRITIS	55	40,38	24,51	3,30			
ESR	RHEUMATOID ARTHRITIS	82	28,61	17,63	1,95	NO—MARGINAL RA HAS HIGHER	1,675	0,096
	OSTEOARTHRITIS	55	23,33	18,79	2,53			
TOTAL CHOLESTEROL	RHEUMATOID ARTHRITIS	82	5,86	1,00	0,11	NO	-0,171	0,864
	OSTEOARTHRITIS	55	5,90	1,31	0,18			
HDL	RHEUMATOID ARTHRITIS	82	1,53	0,37	0,04	YES—RA HAS HIGHER	3,145	0,002
	OSTEOARTHRITIS	55	1,34	0,35	0,05			
LDL	RHEUMATOID ARTHRITIS OSTEOARTHRITIS	82 55	3,74 3,93	0,97 1,32	0,11 0,18	NO	-0,92	0,36
TRIGLYCERIDE	RHEUMATOID ARTHRITIS	82	1,62	0,78	0,09	NO	-1522	0,13
	OSTEOARTHRITIS	55	1,83	0,80	0,11			
CREATININE	RHEUMATOID ARTHRITIS	82	72,01	21,36	2,36	NO	-0,807	0,421
	OSTEOARTHRITIS	55	75,02	21,40	2,89			
BLOOD GLUCOSE	RHEUMATOID ARTHRITIS	82	6,39	3,16	0,35	NO	0,214	0,831
	OSTEOARTHRITIS	55	6,28	2,49	0,34			
HBA1C%	RHEUMATOID ARTHRITIS	82	5,97	1,38	0,15	NO	1,056	0,293
	OSTEOARTHRITIS	55	5,71	1,42	0,19			

*Legend*: *BP* blood pressure, *BMI* body mass index, *HAQ* health assessment questionnaire, *VAS* visual-analog scale, *GH* general health, *ESR* erythrocyte sedimentation rate, *HDL* high density cholesterol, *LDL* low density cholesterol

When comparing CV risk factors in the data of participants who completed the study with available data for the general Croatian population in 2018, we found no significant difference in arterial hypertension after adjusting for standardized age [52].

We observed a significantly higher proportion of smokers in the RA but not in the OA group compared with the general population; of 82 patients with RA, 43 (52.4%) had smoked at least once, and from a sample of 3997 respondents in the population data, 1243 (31.1%) were smokers. The z-test revealed a significant difference between these proportions (z = 4.117, *p* < 0.0001).

Furthermore, of 55 patients with OA, 14 (25.5%) had smoked at least once. The z-test showed no significant difference between the number of smokers in the OA group and representative population data (z = -0.899, *p* = 0.369).

We identified a significantly higher proportion of diabetes in both groups than in general population. In the RA group, 27 (32.9%) of 82 patients had diabetes; in the estimated population of 3 435 425 adults, 284 185 (8.3% or 0.083) had diabetes (z = 8.104, *p* < 0.0001). Further, of 55 patients with OA, 16 (29.1%) had diabetes (z = 5.605, *p* < 0.0001). At final visit HbA1c < 6.5% had 22,2% and 31,25% (*p* = 0.719) for RA and OA group, respectively.

Although the analysis of deceased participants was a secondary, incidental outcome due to the surprisingly high number deaths during study, we performed a detailed investigation, despite the sample size being too small to draw relevant conclusions at the population level. In total, 58 participants died during the study period, 41 with RA and 17 with OA, and the average age was 71.59 years for the RA and 76.96 years for the OA group, with a significantly shorter lifespan for patients with RA. The total incidence of death was higher in the RA group for about one-third of deaths, and the incidence of CVD deaths was also higher. Additionally, the prevalence of modifiable CV risk factors was high in both groups, but not significantly different, except that the RA group had a significantly higher number of smokers and a marginally higher prevalence of increased cholesterol. No association was found between CV death and modifiable risk factors (Tables 8, 9, 10, 11). Table 11 shows the absolute number of deceased participants in both groups, causes of death (CVD and non-CVD), and known risk factors for CVD determined before death. The leading cause of CVD was chronic heart failure, while non-CVD causes included neoplastic diseases at different locations.

**Table 8:** Incidence of death and prevalence of cardiovascular risk factors

Deceased	Rheumatoid arthritis (%)	Osteoarthritis (%)	Total (%)
INCIDENCE OF DECEASED TOTAL	33,10	22,10	28,90
INCIDENCE OF DEATHS FROM CARDIOVASCULAR DISEASE	70,70	58,80	65,50
PREVALENCE OF HYPERTENSION	80,50	70,6	77,60
PREVALENCE OF HYPERCHOLESTEROLEMIA	82,90	70,60	79,30
PREVALENCE OF DIABETES	29,30	41,20	32,80
PREVALENCE OF CIGARETTE SMOKING	36,60	29,40	34,50
MALE GENDER	22,00	17,60	20,70
FEMALE GENDER	78,00	82,40	79,30

**Table 9:** Association of cardiovascular diseases and cardiovascular risk factors for deceased

Statistically significant association	Rheumatoid arthritis	*p*	Osteoarthritis	p
INCIDENCE OF CARDIOVASCULAR DISEASE AND HYPERTENSION	NO	0,237	NO	0,338
INCIDENCE OF CARDIOVASCULAR DISEASE AND HYPERCHOLESTEROLEMIA	NO	0,339	NO	1,000
INCIDENCE OF CARDIOVASCULAR DISEASE AND DIABETES	NO	0,469	NO	1,000
INCIDENCE OF CARDIOVASCULAR DISEASE AND CIGARETTE SMOKING	NO	0,734	NO	0,593
NUMBER OF RISK FACTORS FOR CARDIOVASCULAR AND NON-CARDIOVASCULAR DISEASES	NO	0,289	NO	0,158
MALE AND FEMALE GENDER	NO	1,000	NO	1,000

**Table 10:** Comparison for RA and OA groups for deceased

Comparison of the deceased with rheumatoid arthritis and osteoarthritis	Statistically significant difference	χ2	p
YEARS OF LIFE	YES—RA LIVES SHORTER (t-test)		0,039
PREVALENCE OF HYPERTENSION	NO		0,494
PREVALENCE OF HYPERCHOLESTEROLEMIA	NO		0,307
PREVALENCE OF DIABETES	NO		0,540
PREVALENCE OF CIGARETTE SMOKING	NO		0,764
PREVALENCE OF THE NUMBER OF RISK FACTORS FOR CARDIOVASCULAR DISEASES	NO		0,601
INCIDENCE OF CARDIOVASCULAR DISEASES AS A CAUSE OF DEATH	NO	0,477	0,490

**Table 11:** Association of cardiovascular risk factors with cardiovascular diseases for alive and deceased participants

2016/17 No: RA 123; OA 72	Rheumatoid arthritis—alive	Rheumatoid arthritis—deceased	Osteoart-hritis—alive	Osteoart-hritis—deceased	Statistically significant difference	χ2	p
TOTAL INCIDENCE FOR CARDIOVASCULAR DISEASES FOR RA AND OA (ALIVE + DEAD)	26	28	17	10	NO	0,767	0,381
PREVALENCE OF HYPERTENSION	60	33	44	12	NO	0,118	0,731
PREVALENCE OF DIABETES	27	12	16	7	NO	0,001	0,973
PREVALENCE OF HYPERCHOLESTEROLEMIA	75	34	45	12	NO—MARGINAL RA HAS HIGHER	3,494	0,062
PREVALENCE OF EVER CIGARETTE SMOKING	43	15	14	5	YES—RA HAS HIGHER	8,196	0,004

For subgroup analysis of the age influence participants of both groups were divided into younger and older than 65 years of age. Participants under 65 years in RA group had generally better general health assessment (*p* = 0.001), lower DAS28 (*p* = 0.001), HAQ (*p* = 0.001) and VAS (*p* = 0.02) scores, lower prevalence of hypertension (*p* = 0.001) but higher prevalence of current (*p* = 0.003) and former smokers (*p* = 0.009), lower incidence of CVD (*p* = 0.018) and metabolic syndrome (*p* = 0.004), and marginally lower incidence of heart disease in general (*p* = 0.06). Participants under 65 years in OA group had lower HAQ (*p* = 0.002), increased prevalence of current (*p* = 0.006) and former smokers (*p* = 0.002), trend of lower number of CVD risk factors (*p* = 0.083), and lower incidence of myocardial infarction (0.069). Comparison of equivalent subgroups of the RA and OA groups for those under 65 years showed lower prevalence of general heart disease (0.023) a marginally significant increased incidences of CVD (*p* = 0.09) and stroke (0.054) for RA compared to OA. Comparison of subgroups for those over 65 years, RA showed a significantly higher prevalence of ex-smokers (0.015), and a greater number of risk factors for CVD compared to OA (*p* = 0,018), trends were present in the RA group compared to OA group: higher HAQ (*p* = 0.09), lower overall general health assessment (*p* = 0.09), prevalence of current smokers (*p* = 0.09), incidence of heart failure (*p* = 0.094) and CVD (*p* = 0.079).

Notable results were revealed in the RA subgroup analysis comparing patients who achieved long-term RA remission to those with unsatisfactory inflammatory disease control (Table 12). In the remission group, RA lasted a shorter amount of time, and patients with RA had a significantly lower waist-to-hip ratio, less disability, lower chronic pain score, and better general health assessment. Total cholesterol was higher in the remission group, whereas HDL, plasma glucose, and HbA1c levels were significantly lower. In general, the incidence of CVDs was also significantly lower in the remission group. Additionally, the remission group had a marginally lower BMI, prevalence of arterial hypertension and diabetes, and incidence of heart failure. No differences were observed between groups in the other measured parameters.

**Table 12:** Comparison of participants with remission vs unsatisfactory inflammation control

**Remission vs unsuccessful inflammation control**	**Statistical significance**	**t**	**p**
DISEASE DURATION	YES—FOR REMISSION THE DISEASE LASTS SHORTER	3,815	0,001
BMI	NO—MARGINAL, POOR CONTROL HAS GREATER	1,929	0,058
WAIST TO HIP RATIO	YES—REMISSION HAS LOWER	2,186	0,032
HAQ	YES—REMISSION HAS LOWER	2,861	0,006
VAS	YES—REMISSION HAS LOWER	2,357	0,021
GH	YES—REMISSION HAS BETTER	-2,254	0,028
TOTAL CHOLESTEROL	YES—REMISSION HAS HIGHER	-2125	0,037
HDL	YES- REMISSION HAS GREATER	-2,063	0,043
LDL	NO	-0,663	0,51
TRIGLYCERIDES	NO	-0,413	0,681
CREATININE	NO	-1,459	0,162
BLOOD GLUCOSE	YES—REMISSION HAS LOWER	3,105	0,003
HBA1C	YES—REMISSION HAS LOWER	2,197	0,05
ANTI-CCP	NO	1,378	0,173
**REMISSION VS UNSUCCESSFUL INFLAMMATION CONTROL**	**STATISTICAL SIGNIFICANCE**	**χ2**	**p**
PREVALENCE OF GLUCOCORTICOID USE	YES—REMISSION HAS LESS		0,001
PREVALENCE OF HYPERTENSION	NO—MARGINAL, REMISSION HAS LOWER		0,067
PREVALENCE OF SMOKING EVER	NO	0,993	0,319
PREVALENCE OF SMOKING NOW	NO	0,163	0,687
PREVALENCE OF DIABETES	NO—MARGINAL, REMISSION HAS LOWER	3,608	0,058
PREVALENCE OF HYPERCHOLESTEROLEMIA	NO		1
PREVALENCE OF METABOLIC SYNDROME	NO	1,346	0,246
PREVALENCE OF HEART DISEASE IN GENERAL	NO—MARGINAL, GOOD CONTROL HAS LESS	3,632	0,057
INCIDENCE OF CARDIOVASCULAR DISEASES	NO		0,356
INCIDENCE OF HEART DISEASE—ALL	YES—REMISSION HAS LESS	6,302	0,012
INCIDENCE OF HEART FAILURE	NO—MARGINAL, POOR CONTROL HAS HIGHER		0,56
INCIDENCE OF PERIPHERAL VASCULAR DISEASE	NO		1
INCIDENCE OF TRANSIENT ISCHEMIC ATTACK	NO		0,228
INCIDENCE OF STROKE	NO		0,566
INCIDENCE OF MYOCARDIAL INFARCTION	NO		1
INCIDENCE OF ANGINA PECTORIS	NO		1
INCIDENCE OF CORONARY DISEASE IN OTHER FORMS	NO		1
INCIDENCE OF AORTIC ANEURISM	NO CALCULATIONS		

*Legend*: *BMI* body mass index, *HAQ* health assessment questionnaire, *VAS* visual-analog scale, *GH* general health, *ESR* erythrocyte sedimentation rate, *HDL* high density cholesterol, *LDL* low density cholesterol, *HbA1c* glycated haemoglobin, *anti-CCP* anti-cyclic citrullinated peptides antibody

## Discussion

Assessing CV risk in patients with RA who have an apparently healthy cardiovascular system represents a major challenge, as described in the contemporary ESC and EULAR guidelines [9, 17, 27]. The primary goals of this investigation were to determine the incidence of CV events in the research (RA) group compared with that in the control (OA) group, track the incidence and prevalence of modifiable CV risk factors, and compare the results with known data from the Croatian population. Mortality analysis was performed as a secondary (incidental) assessment because the number of study participants was too small for formal analysis.

Baseline characteristics and differences between RA and OA participants, including a higher prevalence of females in both groups, higher age in the OA group, longer disease duration for RA, a higher proportion of smokers in RA, and higher BMI in OA, are consistent with reported epidemiological and clinical features of the disease [53]. Using OA participants as controls, we tried to minimize two confounding factors: non steroid anti-inflammatory drugs (NSAID) and limitation in physical activity (HAQ) influence. The initial use of NSAIDs was slightly higher in the OA group, and HAQ was higher in the RA group but at the end of investigation difference between both parameters was insignificant.

During the average follow-up period of 8 years, 43.9% of the RA group and 37.5% of the OA group had one or more fatal or non-fatal CV events; of these, 31.7% in the RA and 30.9% in the OA group survived. The incidence was not significantly different between groups, which is in concordance with most previous studies [31, 32, 47, 49], although the patients with RA included in the study were approximately 5 years younger than those with OA. Compared with data from the Croatian population in 2018, a statistically significant difference in CVD deaths was observed for RA but not for OA [52], which may suggest that CV risk estimation or calculators still underestimate risk in patients with RA. According to publications from National Health Institute CVD mortality during investigation years was stable (44–49%) (data exists only for four years). We found comparison of our study mortality to with data from the 2018. to be reasonably representative.

The most common cause of CV events in the present study was chronic heart failure, while acute events occurred much less frequently in both living and deceased participants. This finding differs from that of previous studies in which acute events (fatal and nonfatal) were reported in higher numbers [4, 6, 7]. The total number of deceased patients was surprisingly high for both groups; CVD was cause of death in 70.7% and 58.8% of cases in the RA and OA groups, respectively. Furthermore, the proportion of CV deaths in the RA group was significantly higher than that in the Croatian population [52]; however, the number of patients was too small to have significant power for statistical analysis. Another possible limitation was that only a minority of deceased patients underwent autopsy; other data were obtained from coronary reports by people who were not necessary doctors but did have the medical knowledge to write reports according to Croatian law, and these reports were collected for statistical analysis by the National Health Institute.

Although modifiable CV risk factor analysis was not the primary focus of this study, we found a high prevalence of all modifiable risk factors in both groups with some notable observations. For instance, we identified a much higher prevalence of cigarette smoking in the RA group than in the OA group or general population. Further, all the groups had a similar prevalence of hypertension (after standardization), which is in disagreement with some previous studies [10, 30, 54–58]. We cannot fully explain the higher prevalence of smoking in patients with RA because smoking aggravates RA symptoms, but also may have beneficial psychological effect, especially on those with high stress from chronic disease, or pain. Higher smoker prevalence could have impact on CVD mortality in RA group but in RA subgroup—optimal vs. unsatisfactory inflammation control, smoking prevalence did not differ among groups (*p* = 0.394) while results considering CVD and disk factors did, we can conclude that uncontrolled inflammation was the main contributing factor. We also identified a significantly higher proportion of patients with diabetes in both study groups than in the general population, which may be explained by the influence of chronic inflammation in patients with RA and the older age of the OA group [52, 58–60].

Longitudinal comparison of disease activity for RA showed moderate disease activity throughout investigation with tendency to decrease (4.85 vs. 4.08, t = 4.78, *p* = 0.001), probably do to introduction of more potent therapy.

In comparison of RA subgroup long-term remission with unsatisfactory inflammation control, remission group had a significantly lower incidence of heart diseases in general; marginally lower BMI, prevalence of arterial hypertension, diabetes, and incidence of heart failure. The remission group also had more favorable results for laboratory and other measured parameters. These findings emphasize the need for strict inflammation control and achievement of long-term remission in patients with RA for CV risk control and CVD prevention. This may be explained by the dual nature of the chronic inflammatory effects on the CV system, namely (1) direct effects via activation and promotion of the immune system to cause a cascade of inappropriate immune responses leading to blood vessel damage, atherosclerosis, and thrombosis and (2) indirect effects achieved by promoting or aggravating traditional modifiable CV risk factors, especially hypercholesterolemia (lipid paradox) and diabetes [10, 54, 55, 58–64].

## Conclusion

To cope with the problem of premature CV morbidity and mortality, a multidisciplinary approach for patients with RA is of paramount importance, especially with the cooperation of immunologists and cardiologists for the early detection, prevention, and management of CV risk and diseases. Strict inflammation control plays a central role in achieving these goals and includes developing novel and more effective anti-inflammatory drugs and tightly managing modifiable CV risk factors that contribute to CVD development and aggravate inflammation. Necessary lifestyle modifications include treating hypertension to achieve normal blood pressure values, lowering LDL, controlling diabetes and obesity, ceasing smoking, and adopting other healthy lifestyle habits.

## Data Availability

The databases used or analyzed during the current study are available from the corresponding author on request.

## Abbreviations

RA: Rheumatoid arthritis
CVD: Cardiovascular disease
CV: Cardiovascular
RR: Relative risk
SCORE: Systematic Coronary Risk Evaluation
SCORE 2: Systematic Coronary Risk Evaluation upgraded
SCORE 2 - OP: Systematic Coronary Risk Evaluation upgraded, older person
ESC: European Society of Cardiology
RF: Rheumatoid factor
anti-CCP: Anti-citrulline antibody
EULAR: European Alliance of Associations for Rheumatology
HR: Hazard ratio
MSCT: Multi-slice computed tomography
NSAID: Non steroid anti-inflammatory drugs
OA: Osteoarthritis
ECG: Electrocardiogram
BNP: Brain natriuretic peptide
NT-pro BNP: N-terminal pro-brain natriuretic peptide
STEMI: ST elevated myocardial infarction
NSTEMI: Non-ST elevated myocardial infarction
CRP: C-reactive protein
HDL: High density lipoprotein
LDL: Low density lipoprotein (LDL)
HbA1c: Glycosylated hemoglobin
DAS28-CRP score: Disease activity score in 28 joints using CRP in calculation
